# Have the 10-year outcomes of patients with early inflammatory arthritis improved in the new millennium compared with the decade before? Results from the Norfolk Arthritis Register

**DOI:** 10.1136/annrheumdis-2017-212426

**Published:** 2018-02-23

**Authors:** James M Gwinnutt, Deborah P M Symmons, Alexander J MacGregor, Jacqueline R Chipping, Tarnya Marshall, Mark Lunt, Suzanne M M Verstappen

**Affiliations:** 1 Arthritis Research UK Centre for Epidemiology, Centre for Musculoskeletal Research, Division of Musculoskeletal and Dermatological Sciences, School of Biological Sciences, Faculty of Biology, Medicine and Health, University of Manchester, Manchester Academic Health Science Centre, Manchester, UK; 2 Rheumatology Department, Norfolk and Norwich University Hospitals NHS Trust, Norwich, UK; 3 Norwich Medical School, University of East Anglia Faculty of Medicine and Health Sciences, Norwich, UK; 4 NIHR Manchester Biomedical Research Centre, Central Manchester University Hospitals NHS Foundation Trust, Manchester Academic Health Science Centre, Manchester, UK

**Keywords:** early rheumatoid arthritis, outcomes research, epidemiology, disability, mortality

## Abstract

**Objective:**

To compare the 10-year outcome (disease activity, disability, mortality) of two cohorts of patients with inflammatory polyarthritis (IP) recruited 10 years apart.

**Methods:**

Patients with IP were recruited to the Norfolk Arthritis Register from 1990 to 1994 (cohort 1 (C1)) and from 2000 to 2004 (cohort 2 (C2)). Demographic and clinical data were collected at baseline and at years 1, 2, 3, 5, 7 and 10. Longitudinal disease activity (swollen/tender 51 joint counts (SJC51/TJC51)) and disability (Health Assessment Questionnaire (HAQ)) were compared between the cohorts using population-average negative binomial regression and generalised estimating equation analysis, respectively. Risk of 10-year mortality was compared between cohorts using Cox models. Risk of cardiovascular disease (CVD) mortality was compared between cohorts using competing risks analysis. Mortality rate ratios (MRR), adjusted for changes in mortality risk of the general population, were calculated using Poisson regression.

**Results:**

In total 1653 patients were recruited (C1=1022, C2=631). Patients in C2 had 17% lower SJC51 than C1 over 10 years (95% CI −23% to −10%), whereas TJC51 and HAQ were comparable. C2 patients had reduced risk of all-cause and CVD mortality compared with C1 (all-cause: HR 0.72, 95% CI 0.56 to 0.95; CVD: subhazard ratio 0.58, 95% CI 0.37 to 0.93). After accounting for changes in mortality risk in the general population, the difference in mortality was non-significant (all-cause: MRR 0.78, 95% CI 0.56 to 1.10; CVD: MRR 0.77, 95% CI 0.48 to 1.24).

**Conclusion:**

Disease activity significantly improved in the new millennium, whereas disability and mortality were unchanged.

Inflammatory polyarthritis (IP) and its subset rheumatoid arthritis (RA) are chronic conditions associated with synovial joint inflammation, progressive joint damage and premature mortality.^1 2^ However outcomes can be improved by administration of appropriate therapy.^3 4^ There have been significant advances in the available therapies to treat RA over the past two decades. Methotrexate was introduced as a treatment for RA in the 1990s and became the first-choice synthetic disease-modifying antirheumatic drug (sDMARD).^5 6^ Since then biologic agents (biologic disease-modifying antirheumatic drugs (bDMARDs)) have been introduced and proven to be effective, but costly and are generally not used as first-line therapy.^7 8^ Furthermore there has been a philosophical shift towards treating patients early after symptom onset, which is associated with improved physical function^9–11^ and radiographic damage.^4 12 13^

A number of studies have compared patients during and post the treatment changes of the mid-1990s. An analysis of the Nijmegen early RA cohort compared the 5-year outcome of three subcohorts, based on the time period patients were recruited (subcohort 1=1985–1990, subcohort 2=1990–1995, subcohort 3=1995–2000). Patients in subcohort 3 had significantly lower mean Disease Activity Score (DAS28) at year 5 compared with the other two cohorts (mean DAS28: subcohort 1=3.7, subcohort 2=3.4, subcohort 3=3.2). However the Health Assessment Questionnaire (HAQ) scores were higher at year 5 in the most recent subcohort, although this was not statistically significant (mean HAQ: subcohort 1=0.49, subcohort 2=0.44, subcohort 3=0.83).^14^ Humphreys *et al* compared mortality rates during the first 7 years of follow-up of three cohorts of patients with early RA recruited to the Norfolk Arthritis Register (NOAR) (cohort 1=1990–1994, cohort 2=1995–1999, cohort 3=2000–2004). That analysis reported no significant differences between the mortality rates in each of these cohorts over 7 years of follow-up (mortality rate ratio (MRR): cohort 1=ref, cohort 2=1.13 (95% CI 0.84 to 1.52), cohort 3=1.00 (95% CI 0.70 to 1.43)).^15^

The natural history of IP and RA is becoming less severe^16 17^; therefore, it is difficult to infer whether any improvements in long-term outcome (ie, 10 years) are associated with less severe disease or with the changes in treatment strategy. Thus the aim of this study was to compare the 10-year outcome of two cohorts of patients recruited 10 years apart, controlling for disease severity of the cohorts at baseline. Specifically, the objectives were to compare the baseline and 10-year characteristics of two cohorts of patients, one recruited from 1990 to 1994 and the other from 2000 to 2004, then to compare the disease activity, disability and mortality of the two cohorts over the course of 10 years.

## Patients and methods

The NOAR began recruiting patients with IP registered with a primary care physician (general practitioner (GP)) in the former Norwich Health Authority region, Norfolk, UK in 1990. Incident cases of IP were recruited from GPs or rheumatologists. The inclusion criteria were ≥16 years old and ≥2 swollen joints lasting ≥4 weeks. In this study we included patients recruited from 1990 to 1994 (cohort 1) and those recruited from 2000 to 2004 (cohort 2). Patients were excluded from this analysis if their baseline assessment took place >2 years after symptom onset. Patients were also excluded if they were subsequently diagnosed with a condition other than RA, psoriatic arthritis, postviral arthritis or undifferentiated arthritis. Patients gave written informed consent. More details about NOAR can be found elsewhere.^18^

### Assessments

Patients were assessed at baseline and at 1, 2, 3, 5, 7 and 10 years thereafter. Patients were only assessed beyond year 5 if they had documented swollen joints on two or more occasions or had received disease-modifying antirheumatic drugs (DMARDs) or oral corticosteroids by the fifth year assessment. Demographics were collected at baseline. A research nurse performed a 51 swollen and tender joint count (SJC51/TJC51), from which 28 joint counts were derived. Blood samples were taken at baseline, separated and frozen for future analysis. C reactive protein level (CRP; mg/L), rheumatoid factor positivity (RF; latex test, positive cut-off 40 units/mL) and anticyclic citrullinated peptide antibody positivity (anti-CCP2; tested using the Axis-Shield Diastat Anti-CCP Kit, Dundee, UK; cut-off 5 units/mL) were determined from these samples. The three-component DAS28-CRP was calculated.^19^ The 2010 American College of Rheumatology (ACR)/European League Against Rheumatism (EULAR) criteria for RA were applied retrospectively to the baseline characteristics of the patients.^20^ Patients reported smoking status, and the start and stop dates for all sDMARDs, bDMARDs and oral steroids at each assessment.

### Outcomes

Disease activity was assessed using SJC51 and TJC51 at each assessment, other than assessments 5 and 7 at which joint counts were not performed. Disability was self-reported at each assessment using the HAQ adapted for British use.^21^ This is a validated self-report measure of functional disability that yields a score from 0 (no disability) to 3 (maximum disability). Patients were flagged with the Office for National Statistics (ONS), who provided copies of death certificates as patients died, including date and cause of death, coded using the International Classification of Diseases (ICD) V.9 and V.10. ICD9 codes were recoded to the corresponding ICD10 codes. Initially all-cause mortality was assessed, before assessing death from cancer (ICD10 codes C00–D48), cardiovascular disease (CVD; ICD10 codes I00–I99) or respiratory disease (ICD10 codes J00–J99) as the underlying cause of death. For mortality analysis patients were censored 10 years after symptom onset or on emigration from the country (n=7). Due to ONS flagging, mortality data were complete, regardless of whether patients stopped attending follow-up assessments. The ONS also provided age-specific and sex-specific all-cause and CVD specific mortality rates by calendar year for the Norfolk population (1990–2013) (online supplementary table 1).

10.1136/annrheumdis-2017-212426.supp1 Supplementary file 1

### Statistical analysis

The baseline and 10-year demographic, clinical and treatment characteristics of each cohort were summarised using descriptive statistics. Quantile, logistic or negative binomial regression was used to compare the baseline and 10-year scores between cohorts, depending on the type and distribution of the data. Age at symptom onset and gender were controlled for initially; then other baseline variables were included in the tenth year outcome models (age, gender, symptom duration at baseline, smoking status, SJC51, TJC51, RF, anti-CCP2, CRP, HAQ score and being on sDMARDs/steroids).

To analyse longitudinal disease activity and disability, the median SJC51, TJC51 and HAQ scores over 10 years are displayed using fractional polynomial smoothed plots. Population-average negative binomial regression was used to compare the SJC51 and TJC51 of the two cohorts over the repeated measures. HAQ scores were compared between cohorts over follow-up using generalised estimating equation analysis using the identity link function. All models were initially adjusted for baseline age and gender, before controlling for other baseline characteristics (listed above). Time-varying smoking status was then included in the model to control for differences in prevalence of smoking between the cohorts. Lastly, time-varying DMARD and steroid exposure were included to assess whether differences in medication usage between the two cohorts accounted for any difference in the outcomes. To analyse mortality, Kaplan-Meier survival curves were plotted. A Cox proportional hazards model was used to compare the risk of mortality between cohorts, with the same adjustments made as above. The proportional hazards assumption was met. An MRR was calculated using Poisson regression, which allows comparisons of mortality rates between cohorts, adjusted for the background age-specific and gender-specific mortality rates of the Norfolk population. This model was initially adjusted for baseline age and gender, before adjusting for further baseline characteristics (see above). For this analysis, patients were censored after 10 years of follow-up or at the end of 2013, whichever came first. To analyse risk of specific causes of death, competing risks regression analyses were applied, with each of the three causes of death (cancer, CVD, respiratory disease) as the outcome of interest in turn, with competing risks being other causes of death.^22^ An MRR was also calculated for CVD mortality, using the same methods as above.

Multiple imputation using iterative chained equations was used to account for missing data at assessments which patients attended. Imputed variables were only used as covariates in regression analyses. In sensitivity analysis, we restricted the analyses to those patients fulfilling the 2010 criteria for RA at baseline (n=961). All analyses were performed using Stata V.13.1.

## Results

In total 1653 patients were included in this study: 1022 patients in cohort 1 and 631 in cohort 2 (table 1). Patients in cohort 2 were older at symptom onset and had longer symptom duration at baseline (median (IQR) age (years): cohort 1=54 (41–67), cohort 2=58 (47–70), median difference=4 (95% CI 2 to 6); median (IQR) symptom duration (months): cohort 1=5.1 (2.7–9.4), cohort 2=6.6 (3.9–11.3), median difference=1.5 (95% CI 0.9 to 2.2)). Cohort 2 had lower SJC51 and TJC51 at baseline (median (IQR) SJC51: cohort 1=6 (2–13), cohort 2=3 (1–8), relative difference=−40% (95% CI −46% to −33%); median (IQR) TJC51: cohort 1=7 (3–16), cohort 2=4 (1–12), relative difference −25% (95% CI −33% to −16%)). However, a greater proportion of patients in cohort 2 were on sDMARDs at the time of the baseline assessment (n (%) on sDMARDs at baseline: cohort 1=153 (15.0%), cohort 2=278 (44.1%), OR 4.47 (95% CI 3.54 to 5.65)). Nevertheless patients in cohort 2 had higher functional disability at baseline (median (IQR) HAQ score: cohort 1=0.75 (0.25–1.38), cohort 2=0.88 (0.38–1.63), median difference=0.13 (95% CI 0.01 to 0.24)) (see online supplementary table 2 for the baseline characteristics of patients with RA).

**Table 1:** Baseline characteristics of patients with inflammatory polyarthritis included in the analysis, stratified by cohort

	Cohort 1 (1990–1994)		Cohort 2 (2000–2004)		Median difference/OR/ relative difference (95% CI)*
	N	Median (IQR)	N	Median (IQR)	
Age at symptom onset (years)	1022	54 (41–67)	631	58 (47–70)	4.00 (2.00 to 6.00)
Gender (n (%) female)	662 (64.8)		408 (64.7)		OR 0.99 (0.81 to 1.22)
Smoking status	1021		569		
Never, n (%)	323 (31.6)		181 (31.8)		
Ex-smoker, n (%)	424 (41.5)		245 (43.1)		RRR 1.03 (0.81 to 1.31)
Current smoker, n (%)	274 (26.8)		143 (25.1)		RRR 0.93 (0.71 to 1.22)†
Symptom duration (months)	1022	5.1 (2.7–9.4)	631	6.6 (3.9–11.3)	1.54 (0.89 to 2.18)
Swollen joint counts					
28	1022	5 (1–11)	631	2 (0–6)	− 39% (−45% to −31%)
51	1022	6 (2–13)	631	3 (1–8)	− 40% (−46% to −33%)
Tender joints counts					
28	1022	5 (2–12)	631	2 (0–8)	−29% (−37% to −20%)
51	1022	7 (3–16)	631	4 (1–12)	− 25% (−33% to −16%)
CRP (mg/L)	817	5 (0–16)	521	9.5 (3–22)	4.50 (2.87 to 6.13)
DAS28-CRP	817	3.95 (2.88–5.02)	521	3.60 (2.65–4.53)	−0.35 (−0.56 to −0.15)
HAQ	1010	0.75 (0.25–1.38)	616	0.88 (0.38–1.63)	0.13 (0.01 to 0.24)
RF status	891		553		
Positive, n (%)	252 (28.3)		201 (36.4)		OR 1.45 (1.15 to 1.82)
Anti-CCP2 status	759		511		
Positive, n (%)	178 (23.5)		161 (31.5)		OR 1.50 (1.17 to 1.93)
Met 2010 RA criteria, n (%)	614 (60.1)		347 (55.0)		OR 0.81 (0.66 to 0.99)
Current sDMARDs use, n (%)	153 (15.0)		278 (44.1)		OR 4.47 (3.54 to 5.65)
Treatment delay, months	565	9 (4–22)	471	6 (3–12)	−3.2 (−4.6 to –1.9)

*Quantile/logistic/negative binomial regression was used to compare the two cohorts on each variable depending on the type of variable. Cohort 1 is the reference category.

†Multinomial logistic regression used to compare smoking status between cohorts. Never smoking is the base outcome and cohort 1 is the reference category.

anti-CCP2, anticyclic citrullinated protein antibodies; CRP, C reactive protein; DAS28, Disease Activity Score (28); HAQ, Health Assessment Questionnaire; n, number of patients with available data; RA, rheumatoid arthritis; RF, rheumatoid factor; RRR, relative risk ratio; sDMARD, synthetic disease-modifying antirheumatic drugs.

### Cross-sectional analysis at 10 years

In total 947 (57.3%) patients attended the tenth year assessment (cohort 1=607 (59.4%), cohort 2=340 (53.8%)) (table 2) (see online supplementary table 3 for summary statistics regarding the reasons why patients left the cohort). Patients in cohort 2 had lower SJC51 (median (IQR) SJC51: cohort 1=1 (0–4), cohort 2=0.5 (0–2.5)) compared with cohort 1. After controlling for baseline characteristics, patients in cohort 2 had 33% lower SJC51 compared with cohort 1 at 10 years (95% CI −47% to −15%). The median TJC51 at 10 years were comparable between cohorts, while cohort 2 had a slightly higher median HAQ score; after adjusting for baseline characteristics, the differences were not statistically significant (median (IQR) TJC51: cohort 1=2 (0–11), cohort 2=2 (0–8), relative difference 2% (95% CI −20% to 30%); median (IQR) HAQ: cohort 1=0.88 (0.25–1.63), cohort 2=1.00 (0.25–1.88), median difference −0.01 (−0.16 to 0.14)) (table 2). Similar results were seen when restricting the results to patients with RA who met the 2010 ACR/EULAR criteria (online supplementary table 4).

**Table 2:** Characteristics at 10 years and median change from baseline, stratified by cohort

	Cohort 1 (1990–1994)		Cohort 2 (2000–2004)		Median difference/OR/relative difference (95% CI)*	Median difference/OR/ relative difference (95% CI)†
	N	Median (IQR)	N	Median (IQR)		
Age at 10-year assessment (years)	607	62 (51–72)	340	66 (56–75)	4.00 (1.55 to 6.45)	–
Gender (n (%) female)	422 (69.5)		238 (70.0)		OR 1.02 (0.77 to 1.37)	–
Swollen joint counts (51)	601	1 (0–4)	340	0.5 (0.0–2.5)	−36% (−49% to −20%)	−33% (−47% to −15%)
Median change from baseline	601	−4 (−10 –−1)	340	−2 (−6– 0)		
Tender joints counts (51)	601	2 (0–11)	340	2 (0–8)	−5% (−24% to 19%)	2% (−20% to 30%)
Median change from baseline	601	−3 (−11–1)	340	−1 (−6–2)		
HAQ	597	0.88 (0.25–1.63)	336	1.00 (0.25–1.88)	0.01 (−0.18 to 0.20)	−0.01 (−0.16 to 0.14)
Median change from baseline	588	0.13 (−0.25–0.75)	330	0.00 (−0.38–0.63)		
Current sDMARDs use, n (%)	195 (32.1)		209 (61.5)		OR 3.35 (2.53 to 4.43)	OR 2.71 (1.91 to 3.86)

*Quantile/logistic/negative binomial regression was used to compare the two cohorts on each variable depending on the type of variable. Regressions comparing SJC, TJC, HAQ and current DMARD use between cohorts controlled for age and gender. Cohort 1 is the reference category.

†Quantile/logistic/negative binomial regression used to compare SJC, TJC, HAQ and number on sDMARDs between cohorts at 10 years. These models controlled for baseline: age, gender, symptom duration before baseline, smoking status, SJC (51), TJC (51), RF, anti-CCP, CRP, HAQ score and being on sDMARDs/steroids. Cohort 1 is the reference category.

anti-CCP2, anticyclic citrullinated protein antibodies; CRP, C reactive protein; DMARD, disease-modifying antirheumatic drug; HAQ, Health Assessment Questionnaire; n, number of patients with available data; sDMARD, synthetic disease-modifying antirheumatic drug; SJC, swollen joint count; TJC, tender joint count.

### Longitudinal analysis over 10 years

Consistently more patients in cohort 2 were taking sDMARDs at each follow-up than cohort 1 (table 3). Oral steroid use was also consistently higher in cohort 2 compared with cohort 1. Very few patients in cohort 1 took bDMARDs at any time point as many would have reached their tenth anniversary assessment before bDMARDs were available, whereas 11.8% of patients in cohort 2 were taking bDMARDs at the tenth year follow-up. Data restricted to patients with RA only are shown in online supplementary table 5.

**Table 3:** Number and percentage of patients at each follow-up on different treatments and smoking status, stratified by cohort

	Follow-up assessment						
	0	1	2	3	5	7	10
Patients at assessment							
Cohort 1, n	1022	948	875	832	780	626	607
Cohort 2, n	631	588	530	533	498	407	340
Smoking status							
Cohort 1							
Never, n (%)	323 (31.6)	302 (31.9)	281 (32.1)	265 (31.9)	252 (32.3)	204 (32.6)	193 (31.8)
Ex-smoker, n (%)	424 (41.5)	416 (43.9)	392 (44.8)	385 (46.4)	361 (46.3)	288 (46.0)	287 (47.3)
Current, n (%)	274 (26.8)	229 (24.2)	202 (23.1)	180 (21.7)	167 (21.4)	134 (21.4)	127 (20.9)
Cohort 2							
Never, n (%)	233 (37.1)	218 (37.2)	201 (38.1)	203 (38.2)	195 (39.2)	157 (38.7)	134 (39.5)
Ex-smoker, n (%)	252 (40.1)	244 (41.6)	225 (42.6)	231 (43.5)	211 (42.5)	186 (45.8)	161 (47.5)
Current, n (%)	143 (22.7)	124 (21.2)	102 (19.3)	97 (18.3)	91 (18.3)	63 (15.5)	44 (12.9)
sDMARD*							
Cohort 1, n (%)	153 (15.0)	276 (29.1)	274 (31.3)	269 (32.3)	239 (30.6)	207 (33.1)	195 (32.1)
Cohort 2, n (%)	278 (44.1)	356 (60.5)	327 (61.7)	321 (60.2)	288 (57.8)	256 (62.9)	209 (61.5)
Methotrexate							
Cohort 1, n (%)	12 (1.2)	56 (5.9)	73 (8.3)	93 (11.2)	116 (14.9)	120 (19.8)	129 (21.3)
Cohort 2, n (%)	186 (29.5)	251 (42.7)	231 (43.6)	231 (43.3)	209 (42.0)	196 (48.2)	163 (47.9)
bDMARD*							
Cohort 1, n (%)	0 (0)	0 (0)	0 (0)	0 (0)	0 (0)	1 (0.2)	11 (1.8)
Cohort 2, n (%)	2 (0.3)	5 (0.9)	17 (3.2)	19 (3.6)	27 (5.4)	29 (7.1)	40 (11.8)
Oral steroids							
Cohort 1, n (%)	78 (7.6)	113 (11.9)	109 (12.5)	111 (13.3)	93 (11.9)	70 (11.2)	66 (10.9)
Cohort 2, n (%)	132 (20.9)	124 (21.1)	96 (18.1)	91 (17.1)	83 (16.7)	63 (15.5)	39 (11.5)

Percentages are given as a percentage of the number of patients in the corresponding cohort at the corresponding follow-up.

*sDMARDs included intramuscular gold salts, penicillamine, sulfasalazine, (hydroxy)chloroquine, methotrexate, azathioprine, cyclophosphamide and leflunomide. bDMARDs included etanercept, infliximab, adalimumab and rituximab.

bDMARD, biologic disease-modifying antirheumatic drug; sDMARD, synthetic disease-modifying antirheumatic drug.

**Figure 1:**
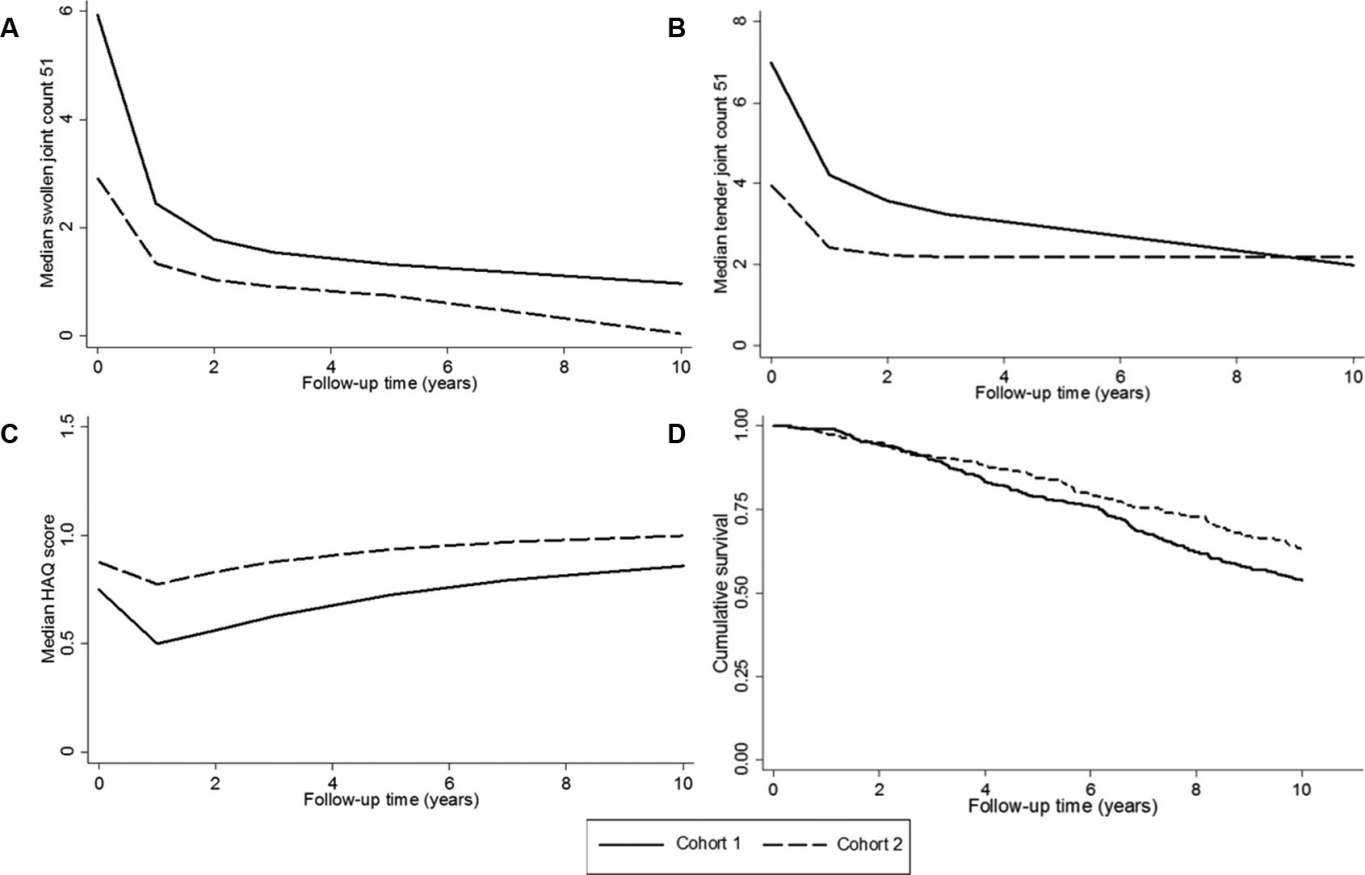
Outcome over 10 years stratified by cohort: (A) median swollen joint count 51, (B) median tender joint count 51, (C) median Health Assessment Questionnaire (HAQ) score and (D) Kaplan-Meier survival curve (adjusted for age and gender (age centred at 70 years)).

Figure 1A,B shows the unadjusted median SJC51 and TJC51 over the course of 10 years, stratified by cohort. Patients in cohort 2 had consistently lower median SJC51 over the course of 10 years and had lower median TJC51 at all follow-ups other than at follow-up 10, at which median TJC51 were comparable. Patients in cohort 2 had, on average, 17% lower SJC51 over 10 years than patients in cohort 1, after adjusting for baseline characteristics (relative difference −17%, 95% CI −23% to −10%), whereas TJC51 were comparable between cohorts over follow-up (relative difference 1%, 95% CI −7% to 10%). At baseline there were 274 current smokers in cohort 1 (26.8%) and 143 in cohort 2 (22.7%). Over the course of follow-up, 78 of these patients quit in cohort 1 (28.5%), whereas 62 quit in cohort 2 (43.4%). However, the inclusion of time-varying smoking status did not substantially alter the results; the inclusion of time-varying treatment also did not alter the results (table 4).

**Table 4:** Comparison of mortality risk, swollen joints, tender joints and functional disability between cohort 1 and cohort 2 over time

	N (number of events)	Age-adjusted and gender-adjusted, HR/SHR (95% CI)	Adjusted for baseline variables*, HR/SHR (95% CI)	Adjusted for baseline variables* and time-varying smoking, HR/SHR (95% CI)	Adjusted for baseline variables* and time-varying treatment/smoking, HR/SHR (95% CI)
Mortality risk†					
Patients with IP	1653 (291)	0.76 (0.60 to 0.96)	0.72 (0.56 to 0.95)	0.73 (0.56 to 0.95)	0.77 (0.58 to 1.02)
Patients with RA	961 (178)	0.87 (0.64 to 1.19)	0.83 (0.59 to 1.18)	0.85 (0.60 to 1.20)	0.95 (0.65 to 1.38)
Cancer mortality risk†					
Patients with IP	1653 (91)	0.97 (0.64 to 1.49)	1.06 (0.66 to 1.73)	1.06 (0.65 to 1.72)	1.04 (0.60 to 1.77)
Patients with RA	961 (55)	1.03 (0.60 to 1.79)	1.25 (0.67 to 2.32)	1.26 (0.67 to 2.35)	1.26 (0.60 to 2.62)
CVD mortality risk†					
Patients with IP	1653 (106)	0.61 (0.40 to 0.93)	0.58 (0.37 to 0.93)	0.53 (0.33 to 0.84)	0.61 (0.37 to 0.99)
Patients with RA	961 (68)	0.69 (0.41 to 1.18)	0.61 (0.34 to 1.12)	0.61 (0.34 to 1.12)	0.79 (0.41 to 1.52)
Respiratory disease mortality risk†					
Patients with IP	1653 (43)	1.26 (0.67 to 2.35)	1.33 (0.67 to 2.66)	1.11 (0.54 to 2.28)	1.01 (0.45 to 2.25)
Patients with RA	961 (25)	1.63 (0.75 to 3.53)	1.50 (0.61 to 3.69)	1.54 (0.64 to 3.72)	1.57 (0.62 to 4.00)

	N	Age-adjusted and gender-adjusted, relative difference (95% CI)	Adjusted for baseline variables*, relative difference (95% CI)	Adjusted for baseline variables* and time-varying smoking, relative difference (95% CI)	Adjusted for baseline variables* and time varing treatment/smoking, relative difference (95% CI)
Swollen joint counts (51)†					
Patients with IP	1653	−34% (−39% to −29%)	−17% (−23% to −10%)	−16% (−23% to −10%)	−15% (−21% to −8%)
Patients with RA	961	−30% (−36% to −23%)	−12% (−20% to −3%)	−11% (−20% to −2%)	−5% (−14% to 6%)
Tender joint counts (51)†					
Patients with IP	1653	−3% (−11% to 5%)	1% (−7% to 10%)	1% (−7% to 10%)	−2% (−10% to 7%)
Patients with RA	961	0% (−10% to 12%)	−4% (−14% to 7%)	−4% (−14% to 8%)	−5% (−15% to 6%)

	N	Age-adjusted and gender-adjusted, mean difference (95% CI)	Adjusted for baseline variables*, mean difference (95% CI)	Adjusted for baseline variables* and time-varing smoking, mean difference (95% CI)	Adjusted for baseline variables* and time-varying treatment/smoking, mean difference (95% CI)
HAQ scores†					
Patients with IP	1651	0.09 (0.03 to 0.16)	0.05 (−0.00 to 0.10)	0.05 (−0.00 to 0.10)	0.01 (−0.05 to 0.06)
Patients with RA	960	0.08 (−0.01 to 0.17)	0.01 (−0.06 to 0.08)	0.01 (−0.06 to 0.08)	−0.05 (−0.12 to 0.02)

Cohort 1 is the reference category for all models.

*Baseline variables controlled for age, gender, time from onset to baseline, rheumatoid factor, anticyclic citrullinated protein antibodies, smoking status, HAQ, swollen/tender joint counts (51), C reactive protein, taking sDMARDs and Disease Activity Score 28.

†Mortality risk modelled using Cox proportional hazards model, cancer/CVD/respiratory disease modelled using competing risks regression, swollen and tender joint counts modelled using population-average negative binomial regression, and HAQ score modelled using generalised estimating equations analysis.

CVD, cardiovascular disease; HAQ, Health Assessment Questionnaire; IP, inflammatory polyarthritis; RA, rheumatoid arthritis; sDMARD, synthetic disease-modifying antirheumatic drug; SHR, subhazard ratio.

Figure 1C displays the median HAQ score over the course of 10 years, stratified by cohort. Patients in cohort 2 had on average 0.09 higher HAQ score over follow-up compared with cohort 1 (95% CI 0.03 to 0.16), after controlling for age and gender. However after controlling for baseline variables, the HAQ scores between the cohorts over 10 years were comparable (table 4). The same was true after including time-varying smoking and treatment into the model.

During 15 185 person-years of follow-up, 291 patients died (cohort 1=179 (17.5%), cohort 2=112 (17.8%)). Figure 1D shows the Kaplan-Meier curves for the two cohorts. Patients in cohort 2 had reduced risk of mortality compared with cohort 1 after adjusting for age and gender (HR 0.76, 95% CI 0.60 to 0.96) and after adjusting for baseline variables (HR 0.72, 95% CI 0.56 to 0.95). However, there was no significant difference in risk of mortality between the cohorts when restricting the analysis to patients who met the 2010 ACR/EULAR RA criteria at baseline (table 4). Including time-varying smoking and treatment into the model did not substantially alter the results (HR 0.77, 95% CI 0.58 to 1.02). The MRR for cohort 2 compared with cohort 1 was 0.96 (95% CI 0.75 to 1.22) after adjusting for age and gender. After further adjustment, the MRR was 0.78 (95% CI 0.56 to 1.10), indicating no significant difference in mortality in cohort 2 after accounting for differences in the background risk of death between the cohorts.

The proportion of patients dying from CVD was lower in cohort 2 than in cohort 1 (CVD n (% total died): cohort 1=72 (40.2%), cohort 2=34 (30.4%)), and this was statistically significant, after adjusting for age and gender (subhazard ratio (SHR) 0.61, 95% CI 0.40 to 0.93) and after adjusting for baseline variables (SHR 0.58, 95% CI 0.37 to 0.93). Adjustment for time-varying smoking and time-varying treatment did not substantially alter the estimate (SHR 0.61 (95% CI 0.37 to 0.99)). The MRR for CVD mortality was 0.90 (95% CI 0.59, 1.37) when adjusting for age and gender. After further adjustment, the MRR was 0.77 (95% CI 0.48 to 1.24), indicating no significant difference in CVD mortality in cohort 2 compared with cohort 1, over the secular change of background risk of CVD mortality in the general population. The proportions of patients dying from cancer and respiratory disease were slightly higher in cohort 2 (cancer, n (% total died): cohort 1=53 (29.6%), cohort 2=38 (33.9%); respiratory disease, n (% total died): cohort 1=22 (12.3%), cohort 2=21 (18.7%)), and the risk of death from these causes was not significantly different between the two cohorts (table 4).

## Discussion

This analysis shows that patients with early IP with symptom onset in the new millennium had lower 10-year mortality risk than patients with symptom onset 10 years earlier. However, after controlling for the background mortality risk in the general population, the difference in mortality risk between the cohorts was non-significant. In addition, SJCs were significantly lower in patients with IP in cohort 2 compared with cohort 1. However, TJC and disability over 10 years were not improved for patients in cohort 2 compared with cohort 1.

A meta-analysis of 11 longitudinal studies of patients with RA reported decreasing mortality rate from 1955 to 1995.^23^ Furthermore, a recent analysis of data from 31 countries assessing mortality with RA as the underlying cause reported a reduction in mortality rates over the period between 1987 and 2011 (mean pooled age-standardised rate: 1987–1989=7.1/million person-years, 2009–2011=3.7/million person-years).^24^ However, our analysis did not demonstrate a significant reduction in 10-year mortality in patients with early IP, after accounting for secular changes in the background mortality risk of the general population. Our results are in line with a study of patients with RA from Ontario, Canada, which reported no significant change in MRR over the period 1996–2009.^25^

A recent analysis from a cohort of patients recruited in Olmsted County, Minnesota, reported a reduction in CVD mortality over a 10-year period for patients recruited from 2000 to 2007 compared with patients recruited from 1990 to 1999.^26^ While the effect estimate from our analysis illustrated a reduction in CVD mortality, the CI overlapped 1, meaning our analysis cannot confirm the conclusions of the Olmsted County study.

The results of the disease activity and disability analyses extend previous literature looking at outcome of patients over 5 years.^14^ However, our analysis is the first study to directly compare the longitudinal clinical outcome over 10 years between two cohorts of patients recruited 10 years apart. Including treatment variables as covariates tempered the association between cohort inclusion and long-term disability and SJC51, but disability remained comparable between cohorts and SJC51 remained significantly lower for cohort 2 compared with cohort 1. While this suggests that there may be factors other than treatment influencing the association between cohort and outcome, it is possible that there is residual confounding by treatment given and treatment response as we were only able to adjust for whether or not the patient was on a DMARD at each assessment.

Different patterns of device usage between the cohorts could be influencing the HAQ scores. However, recalculating the HAQ to remove the device adjustment did not alter the results (data not shown). An explanation for why there were no improvements in ‘subjective’ outcomes (TJC, HAQ) could be that the expectation of treatment efficacy was higher in patients in cohort 2 compared with cohort 1. Thus a similar level of disability may be rated as higher in cohort 2 compared with cohort 1.^27^ A qualitative study published in 2004 reported that some patients had very high expectations of the efficacy of treatment,^28^ and expectations of patients have been demonstrated to be associated with differences in pain following joint replacement surgery^29 30^ and adherence to medication.^31^

This study has a number of strengths. The continuous recruitment and standardised assessment of patients in NOAR over 20 years meant that we were able to compare the long-term outcomes of patients treated in different treatment eras. Adjusting for the baseline characteristics of the patients controlled for the secular change towards reduced severity of IP and RA at presentation.^16 17^ As with any study following patients over an extended period, there was attrition in both cohorts. However the characteristics of the patients leaving the study did not differ between cohorts, and therefore attrition is not biasing the analysis. Furthermore, as sDMARD dose was not routinely collected for these cohorts, we were unable to analyse changes in dose over follow-up.

In conclusion we have shown a 17% reduction in SJCs in patients with more recent symptom onset. However the 10-year mortality, TJCs and functional disability of patients did not significantly differ between the cohorts.
