# Supplementary material for: Have the 10-year outcomes of patients with early inflammatory arthritis improved in the new millennium compared with the decade before? Results from the Norfolk Arthritis Register
Source: Ann Rheum Dis. 2018 Feb 23;77(6):848–54. doi: 10.1136/annrheumdis-2017-212426 (PMC5965352; doi:10.1136/annrheumdis-2017-212426)
Supplement: Supplementary file 1 [file annrheumdis-2017-212426supp001.docx]

| *Supplementary Table 1 – Number of deaths and total population for each ten year age band of the Norfolk population (1990-2013), stratified by gender* | 1990 | | 1991 | | 1992 | | 1993 | | 1994 | | 1995 | |
| --- | --- | --- | --- | --- | --- | --- | --- | --- | --- | --- | --- | --- |
| Age band | Deaths | Population (1000s) | Deaths | Population (1000s) | Deaths | Population (1000s) | Deaths | Population (1000s) | Deaths | Population (1000s) | Deaths | Population (1000s) |
| 15-24 |  |  |  |  |  |  |  |  |  |  |  |  |
| M | 52 | 51.9 | 35 | 51.6 | 46 | 50.3 | 37 | 48.4 | 45 | 46.5 | 43 | 45.0 |
| F | 9 | 49.2 | 12 | 48.7 | 14 | 47.4 | 17 | 45.7 | 16 | 44.5 | 17 | 43.6 |
| 25-34 |  |  |  |  |  |  |  |  |  |  |  |  |
| M | 70 | 50.1 | 45 | 50.7 | 55 | 41.5 | 54 | 51.9 | 59 | 52.5 | 55 | 53.0 |
| F | 29 | 49.9 | 14 | 50.6 | 23 | 50.7 | 26 | 50.9 | 20 | 51.7 | 25 | 52.0 |
| 35-44 |  |  |  |  |  |  |  |  |  |  |  |  |
| M | 89 | 51.7 | 74 | 51.7 | 67 | 50.1 | 74 | 49.0 | 63 | 48.5 | 76 | 48.4 |
| F | 56 | 52.3 | 44 | 52.6 | 60 | 50.7 | 36 | 50.0 | 48 | 50.1 | 45 | 49.8 |
| 45-54 |  |  |  |  |  |  |  |  |  |  |  |  |
| M | 167 | 44.2 | 175 | 45.5 | 172 | 48.2 | 205 | 50.0 | 152 | 51.1 | 174 | 52.3 |
| F | 109 | 43.3 | 134 | 44.7 | 100 | 47.6 | 125 | 49.7 | 108 | 51.1 | 117 | 52.5 |
| 55-64 |  |  |  |  |  |  |  |  |  |  |  |  |
| M | 467 | 40.0 | 485 | 40.2 | 419 | 40.4 | 449 | 40.8 | 403 | 41.2 | 411 | 41.6 |
| F | 300 | 42.5 | 280 | 42.5 | 247 | 42.4 | 274 | 42.4 | 270 | 42.7 | 264 | 42.8 |
| 65-74 |  |  |  |  |  |  |  |  |  |  |  |  |
| M | 1148 | 36.3 | 1151 | 36.9 | 1217 | 37.5 | 1169 | 38.3 | 1251 | 39.1 | 1143 | 38.5 |
| F | 797 | 42.1 | 812 | 42.4 | 769 | 43.0 | 795 | 44.1 | 773 | 44.8 | 812 | 44.1 |
| 75-84 |  |  |  |  |  |  |  |  |  |  |  |  |
| M | 1768 | 19.8 | 1756 | 20.0 | 1641 | 20.0 | 1652 | 19.6 | 1611 | 19.3 | 1659 | 20.4 |
| F | 1673 | 30.2 | 1661 | 30.5 | 1629 | 30.3 | 1688 | 29.7 | 1580 | 29.0 | 1541 | 30.2 |
| 85+ |  |  |  |  |  |  |  |  |  |  |  |  |
| M | 723 | 3.7 | 815 | 3.9 | 787 | 4.1 | 904 | 4.4 | 829 | 4.6 | 928 | 4.9 |
| F | 1595 | 9.7 | 1632 | 10.0 | 1629 | 10.4 | 1759 | 10.9 | 1688 | 11.3 | 1919 | 11.8 |

F=female, M=male

| *Supplementary Table 1 (cont.) – Number of deaths and total population for each ten year age band of the Norfolk population (1990-2013), stratified by gender* | 1996 | | 1997 | | 1998 | | 1999 | | 2000 | | 2001 | |
| --- | --- | --- | --- | --- | --- | --- | --- | --- | --- | --- | --- | --- |
| Age band | Deaths | Population (1000s) | Deaths | Population (1000s) | Deaths | Population (1000s) | Deaths | Population (1000s) | Deaths | Population (1000s) | Deaths | Population (1000s) |
| 15-24 |  |  |  |  |  |  |  |  |  |  |  |  |
| M | 20 | 44.0 | 36 | 42.8 | 54 | 42.2 | 29 | 41.8 | 33 | 42.7 | 35 | 44.0 |
| F | 17 | 42.7 | 11 | 42.0 | 14 | 41.7 | 11 | 41.2 | 19 | 41.6 | 8 | 42.6 |
| 25-34 |  |  |  |  |  |  |  |  |  |  |  |  |
| M | 54 | 53.4 | 54 | 53.7 | 60 | 53.7 | 53 | 52.4 | 52 | 51.1 | 51 | 49.1 |
| F | 24 | 52.6 | 29 | 53.0 | 29 | 53.0 | 14 | 52.2 | 27 | 51.2 | 18 | 49.5 |
| 35-44 |  |  |  |  |  |  |  |  |  |  |  |  |
| M | 59 | 49.0 | 61 | 49.9 | 72 | 51.0 | 70 | 52.1 | 77 | 53.4 | 80 | 54.7 |
| F | 45 | 50.2 | 47 | 50.9 | 57 | 51.5 | 42 | 52.5 | 42 | 53.9 | 42 | 54.8 |
| 45-54 |  |  |  |  |  |  |  |  |  |  |  |  |
| M | 173 | 53.4 | 176 | 53.9 | 192 | 54.1 | 193 | 54.4 | 175 | 54.3 | 169 | 53.9 |
| F | 145 | 53.8 | 124 | 54.6 | 142 | 55.2 | 161 | 55.8 | 124 | 56.1 | 141 | 56.1 |
| 55-64 |  |  |  |  |  |  |  |  |  |  |  |  |
| M | 426 | 41.8 | 359 | 42.4 | 400 | 43.5 | 407 | 45.3 | 432 | 47.0 | 413 | 48.8 |
| F | 233 | 42.9 | 219 | 43.3 | 240 | 44.5 | 248 | 46.4 | 282 | 48.1 | 250 | 49.9 |
| 65-74 |  |  |  |  |  |  |  |  |  |  |  |  |
| M | 1162 | 38.2 | 1024 | 38.2 | 1014 | 38.3 | 1070 | 38.7 | 979 | 39.2 | 903 | 39.6 |
| F | 698 | 43.7 | 758 | 43.4 | 750 | 42.9 | 684 | 42.9 | 612 | 43.0 | 611 | 43.3 |
| 75-84 |  |  |  |  |  |  |  |  |  |  |  |  |
| M | 1619 | 21.3 | 1609 | 22.1 | 1778 | 22.7 | 1668 | 22.9 | 1647 | 23.3 | 1633 | 24.1 |
| F | 1577 | 31.0 | 1535 | 31.7 | 1506 | 32.3 | 1603 | 32.6 | 1510 | 32.7 | 1498 | 33.1 |
| 85+ |  |  |  |  |  |  |  |  |  |  |  |  |
| M | 958 | 5.1 | 983 | 5.3 | 1028 | 5.6 | 1095 | 5.7 | 1120 | 5.9 | 1112 | 6.1 |
| F  F=female, M=male | 1902 | 12.2 | 1910 | 12.5 | 2003 | 13.0 | 2083 | 13.2 | 2037 | 13.6 | 2093 | 14.1 |

| *Supplementary Table 1 (cont.) – Number of deaths and total population for each ten year age band of the Norfolk population (1990-2013), stratified by gender* | 2002 | | 2003 | | 2004 | | 2005 | | 2006 | | 2007 | |
| --- | --- | --- | --- | --- | --- | --- | --- | --- | --- | --- | --- | --- |
| Age band | Deaths | Population (1000s) | Deaths | Population (1000s) | Deaths | Population (1000s) | Deaths | Population (1000s) | Deaths | Population (1000s) | Deaths | Population (1000s) |
| 15-24 |  |  |  |  |  |  |  |  |  |  |  |  |
| M | 33 | 45.4 | 26 | 46.6 | 38 | 47.5 | 40 | 48.6 | 41 | 49.8 | 23 | 50.9 |
| F | 15 | 43.6 | 15 | 45.0 | 16 | 46.0 | 14 | 46.7 | 14 | 47.5 | 9 | 48.9 |
| 25-34 |  |  |  |  |  |  |  |  |  |  |  |  |
| M | 56 | 48.1 | 46 | 47.0 | 50 | 45.8 | 51 | 45.4 | 44 | 45.2 | 48 | 45.0 |
| F | 30 | 48.1 | 21 | 47.1 | 20 | 46.3 | 17 | 45.9 | 19 | 45.5 | 8 | 45.2 |
| 35-44 |  |  |  |  |  |  |  |  |  |  |  |  |
| M | 82 | 55.9 | 70 | 56.6 | 74 | 57.0 | 85 | 57.6 | 90 | 57.8 | 86 | 57.4 |
| F | 50 | 55.7 | 48 | 56.5 | 37 | 57.4 | 55 | 57.9 | 62 | 58.3 | 59 | 58.5 |
| 45-54 |  |  |  |  |  |  |  |  |  |  |  |  |
| M | 164 | 52.6 | 163 | 51.9 | 159 | 51.7 | 145 | 52.0 | 176 | 52.6 | 140 | 53.4 |
| F | 142 | 54.7 | 130 | 54.0 | 107 | 53.9 | 117 | 54.0 | 95 | 54.5 | 115 | 55.3 |
| 55-64 |  |  |  |  |  |  |  |  |  |  |  |  |
| M | 443 | 51.3 | 429 | 53.4 | 413 | 54.8 | 407 | 56.0 | 418 | 57.2 | 445 | 57.8 |
| F | 269 | 52.8 | 284 | 55.1 | 304 | 56.8 | 303 | 58.2 | 271 | 59.4 | 279 | 60.6 |
| 65-74 |  |  |  |  |  |  |  |  |  |  |  |  |
| M | 914 | 40.1 | 904 | 40.8 | 835 | 41.4 | 884 | 41.9 | 799 | 42.2 | 790 | 42.8 |
| F | 618 | 43.7 | 595 | 43.9 | 593 | 44.4 | 519 | 44.7 | 542 | 44.7 | 563 | 44.9 |
| 75-84 |  |  |  |  |  |  |  |  |  |  |  |  |
| M | 1762 | 24.6 | 1599 | 25.3 | 1624 | 25.9 | 1675 | 52.6 | 1506 | 26.5 | 1504 | 26.8 |
| F | 1599 | 33.5 | 1532 | 34.3 | 1545 | 34.7 | 1404 | 34.5 | 1497 | 34.3 | 1402 | 34.4 |
| 85+ |  |  |  |  |  |  |  |  |  |  |  |  |
| M | 1129 | 6.1 | 1109 | 6.0 | 1087 | 6.3 | 1273 | 6.7 | 1247 | 7.3 | 1224 | 7.7 |
| F  F=female, M=male | 2194 | 14.1 | 2199 | 13.9 | 2129 | 13.7 | 2203 | 14.5 | 2171 | 15.2 | 2224 | 15.9 |

| *Supplementary Table 1 (cont.) – Number of deaths and total population for each ten year age band of the Norfolk population (1990-2013), stratified by gender* | 2008 | | 2009 | | 2010 | | 2011 | | 2012 | | 2013 | |
| --- | --- | --- | --- | --- | --- | --- | --- | --- | --- | --- | --- | --- |
| Age band | Deaths | Population (1000s) | Deaths | Population (1000s) | Deaths | Population (1000s) | Deaths | Population (1000s) | Deaths | Population (1000s) | Deaths | Population (1000s) |
| 15-24 |  |  |  |  |  |  |  |  |  |  |  |  |
| M | 33 | 52.6 | 29 | 53.1 | 20 | 54.2 | 24 | 52.1 | 21 | 52.1 | 25 | 52.2 |
| F | 15 | 49.9 | 7 | 50.5 | 10 | 50.3 | 7 | 50.4 | 4 | 50.3 | 12 | 50.5 |
| 25-34 |  |  |  |  |  |  |  |  |  |  |  |  |
| M | 40 | 46.0 | 41 | 47.0 | 37 | 49.2 | 44 | 48.4 | 33 | 49.3 | 44 | 50.1 |
| F | 20 | 45.1 | 29 | 45.4 | 18 | 46.6 | 21 | 47.3 | 15 | 48.3 | 27 | 49.3 |
| 35-44 |  |  |  |  |  |  |  |  |  |  |  |  |
| M | 100 | 57.1 | 106 | 56.3 | 85 | 55.2 | 58 | 53.5 | 82 | 52.1 | 75 | 50.9 |
| F | 48 | 58.1 | 39 | 57.4 | 41 | 56.0 | 45 | 54.5 | 45 | 53.2 | 45 | 51.9 |
| 45-54 |  |  |  |  |  |  |  |  |  |  |  |  |
| M | 159 | 54.3 | 165 | 55.2 | 185 | 56.6 | 141 | 57.1 | 164 | 58.1 | 171 | 59.0 |
| F | 117 | 55.9 | 119 | 56.8 | 110 | 57.9 | 111 | 58.7 | 106 | 59.4 | 121 | 60.3 |
| 55-64 |  |  |  |  |  |  |  |  |  |  |  |  |
| M | 432 | 57.7 | 421 | 57.4 | 409 | 57.2 | 384 | 55.8 | 399 | 53.9 | 328 | 53.1 |
| F | 301 | 61.1 | 274 | 61.0 | 301 | 60.8 | 279 | 60.0 | 257 | 58.1 | 268 | 57.0 |
| 65-74 |  |  |  |  |  |  |  |  |  |  |  |  |
| M | 803 | 44.1 | 811 | 45.4 | 781 | 46.8 | 790 | 47.7 | 815 | 50.4 | 811 | 52.3 |
| F | 573 | 46.1 | 563 | 47.4 | 529 | 48.9 | 584 | 49.6 | 559 | 52.8 | 617 | 55.0 |
| 75-84 |  |  |  |  |  |  |  |  |  |  |  |  |
| M | 1538 | 27.3 | 1439 | 27.8 | 1458 | 28.8 | 1430 | 28.6 | 1456 | 29.4 | 1526 | 30.2 |
| F | 1365 | 34.4 | 1268 | 34.4 | 1299 | 34.6 | 1252 | 35.0 | 1291 | 35.3 | 1253 | 35.6 |
| 85+ |  |  |  |  |  |  |  |  |  |  |  |  |
| M | 1324 | 8.2 | 1340 | 8.5 | 1360 | 8.9 | 1390 | 8.9 | 1456 | 9.2 | 1529 | 9.5 |
| F  F=female, M=male | 2283 | 16.3 | 2355 | 16.6 | 2304 | 16.9 | 2255 | 17.1 | 2343 | 17.5 | 2505 | 17.8 |

|  | Cohort 1 (1990-94) | | Cohort 2 (2000-04) | |  |
| --- | --- | --- | --- | --- | --- |
|  | N | Median (IQR) | N | Median (IQR) | Median difference / OR / relative difference (95% CI)* |
| Age at symptom onset (years) | 614 | 56 (44, 68) | 347 | 59 (49, 69) | 3.00 (0.32 to 5.68) |
| Gender (N (%) female) | 404 (65.8) |  | 238 (68.6) |  | 1.13 (0.86 to 1.60) |
| Smoking status:  Never, N (%)  Ex-smoker, N (%)  Current smoker, N (%) | 614  182 (29.6)  260 (42.4)  172 (28.0) |  | 310  93 (30.0)  137 (44.2)  80 (25.8) |  | RRR 1.03 (0.75 to 1.43)  RRR 0.91 (0.63 to 1.31) *§* |
| Symptom duration (months) | 614 | 5.3 (2.9, 9.9) | 347 | 7.0 (4.5, 11.8) | 1.71 (0.91 to 2.50) |
| Swollen joint counts:  28  51 | 614  614 | 9 (4, 14)  11 (6, 17) | 347  347 | 5 (2, 10)  6 (3, 12) | -34% (-41% to -26%)  -35% (-42% to -28%) |
| Tender joints counts:  28  51 | 614  614 | 10 (4, 16)  14 (7, 23) | 347  347 | 6 (2, 14)  10 (3, 19) | -22% (-31% to -13%)  -18% (-27% to -8%) |
| CRP (mg/l) | 518 | 8 (1, 22) | 309 | 13 (5, 26) | 4.60 (2.24 to 6.96) |
| DAS28-CRP | 518 | 4.59 (3.83, 5.57) | 309 | 4.27 (3.52, 4.95) | -0.32 (-0.56 to -0.09) |
| HAQ | 608 | 1.06 (0.50, 1.63) | 340 | 1.13 (0.63, 1.88) | 0.00 (-0.15to 0.15) |
| RF status:  Positive, N (%) | 559  224 (40.1) |  | 316  166 (52.5) |  | OR 1.66 (1.25 to 2.19) |
| Anti-CCP2 status:  Positive, N (%) | 462  167 (36.2) |  | 288  132 (45.8) |  | OR 1.49 (1.11 to 2.02) |
| Current sDMARDs use, N (%) | 120 (19.5) |  | 187 (53.9) |  | OR 4.67 (3.49 to 6.23) |

*Supplementary Table 2 – Baseline characteristics of the patients who met the 2010 ACR/EULAR RA criteria at baseline*

** Quantile / logistic / negative binomial regression were used to compare the two cohorts on each variable depending on the type of variable. Cohort 1 is the reference category.*

*§ multinomial logistic regression used to compare smoke status between cohorts. Never smoking is the base outcome and Cohort 1 is the reference category.*

*Anti-CCP2 = Anti-citrullinated protein antibodies,* coef. *= regression coefficient, DAS28 = Disease activity score (28), HAQ = Health assessment questionnaire, IQR = Interquartile range, IRR = incident rate ratio, l = Litres, mg = Milligrams, N = Number of patients with available data, OR = odds ratio, RA = Rheumatoid arthritis RF = Rheumatoid factor, RRR = relative risk ratio, sDMARD = Synthetic Disease Modifying Anti-Rheumatic Drugs*

*Supplementary Table 3 – Reasons patients left the cohort over 10 years*

| Reason patients left the cohort | Cohort 1, N(%) | Cohort 2, N(%) |
| --- | --- | --- |
| Total | 415 | 291 |
| Death § | 148 (35.7) | 93 (32.0) |
| Declining further follow-up / lost to follow-up | 214 (51.6) | 144 (49.5) |
| Becoming ineligible after the 5^th^ assessment | 22 (5.3) | 26 (8.9) |
| Missing 10^th^ anniversary assessment but still engaged with NOAR  *§ Patients may have left the cohort for a different reason and the subsequently died* | 31 (7.5) | 28 (9.6) |

*Supplementary Table 4 – Characteristics at 10 years and median change from baseline, stratified by cohort (including only patients who met the 2010 ACR/EULAR RA criteria at baseline)*

|  | Cohort 1: 1990-94 | | Cohort 2: 2000-04 | |  |  |
| --- | --- | --- | --- | --- | --- | --- |
|  | N | Median (IQR) | N | Median (IQR) | Median difference / OR / relative difference (95% CI)* | Median difference / OR / relative difference (95% CI)§ |
| Age at ten year assessment (years) | 381 | 63 (53, 74) | 200 | 66 (59, 76) | 3.34 (1.05 to 5.63) | - |
| Gender (N (%) female) | 268 (70.3) |  | 148 (74.0) |  | 1.20 (0.82 to 1.76) | - |
| Swollen joint counts (51):  Median change from baseline | 377  377 | 2 (0, 5)  -7 (-14, -2) | 200  200 | 1 (0, 3)  -4 (-9, -1) | -39% (-53% to 19.4%) | -33% (-48.7 to -11.3) |
| Tender joints counts (51):  Median change from baseline | 377  377 | 3 (1, 12)  -7 (-16, 0) | 200  200 | 2 (0, 11)  -3 (-12, 1) | -2% (-24% to 28%) | -2% (-27% to 31%) |
| HAQ  Median change from baseline | 373  368 | 1.25 (0.38, 2.00)  0.13 (-0.25, 0.81) | 197  194 | 1.25 (0.50, 2.00)  0.00 (-0.38, 0.50) | -0.08 (-0.22 to 0.06) | -0.17 (-0.32 to -0.02) |
| Current sDMARDs use, N (%) | 165 (43.3) |  | 152 (76.0) |  | OR 4.31 (2.93 to 6.36) | OR 2.80 (1.85 to 4.26) |

** Quantile / logistic / negative binomial regression were used to compare the two cohorts on each variable depending on the type of variable. Regressions comparing SJC, TC, HAQ and current DMARD use between cohorts controlled for age and gender, other than when age or gender were the independent variable in which the case the analysis was univariate. Cohort 1 is the reference category.*

*§ Quantile / logistic / negative binomial regression used to compare SJC, TJC, HAQ and number on sDMARDs between cohorts at ten years. These models controlled for baseline: age, gender, symptom duration before baseline, smoking status, swollen joint count (51), tender joint count (51), RF, anti-CCP2, CRP, HAQ score, and being on sDMARDs/steroids. Cohort 1 is the reference category.*

*CI = confidence interval, coef. = regression coefficient, HAQ = Health assessment questionnaire, IQR = Interquartile range, IRR = incident rate ratio, N = Number of patients with available data, OR = Odds ratio, sDMARD = Synthetic Disease Modifying Anti-Rheumatic Drugs*

*Supplementary Table 5 – Number and percentage of 2010 ACR/EULAR RA patients at each follow-up on different treatments and smoking status, stratified by cohort*

|  | Follow-up assessment | | | | | | |
| --- | --- | --- | --- | --- | --- | --- | --- |
|  | **0** | **1** | **2** | **3** | **5** | **7** | **10** |
| Patients at assessment |  |  |  |  |  |  |  |
| Cohort 1, N | 614 | 583 | 538 | 515 | 478 | 396 | 381 |
| Cohort 2, N | 347 | 330 | 307 | 303 | 284 | 244 | 200 |
| Smoking Status |  |  |  |  |  |  |  |
| Cohort 1: |  |  |  |  |  |  |  |
| Never, N (%) | 182 (29.6) | 173 (29.7) | 163 (30.3) | 154 (29.9) | 146 (30.5) | 121 (30.6) | 114 (29.9) |
| Ex-Smoker, N (%) | 260 (42.4) | 263 (45.1) | 248 (46.1) | 246 (47.8) | 226 (47.3) | 189 (47.7) | 188 (49.3) |
| Current, N (%) | 172 (28.0) | 147 (25.2 | 127 (23.6) | 115 (22.3) | 106 (22.2) | 86 (21.7) | 79 (20.7) |
| Cohort 2: |  |  |  |  |  |  |  |
| Never, N (%) | 127 (36.6) | 123 (37.3) | 115 (37.5) | 117 (38.6) | 114 (40.1) | 94 (38.5) | 79 (39.5) |
| Ex-Smoker, N (%) | 140 (40.4) | 137 (41.5) | 129 (42.0) | 130 (42.9) | 117 (41.2) | 112 (45.9) | 91 (45.5) |
| Current, N (%) | 80 (23.1) | 70 (21.2) | 63 (20.5) | 56 (18.5) | 53 (18.7) | 38 (15.6) | 30 (15.0) |
| sDMARD* |  |  |  |  |  |  |  |
| Cohort 1, N (%) | 120 (19.5) | 214 (36.7) | 215 (40.0) | 216 (41.9) | 185 (38.7) | 162 (40.9) | 159 (41.7) |
| Cohort 2, N (%) | 186 (53.6) | 236 (71.5) | 223 (72.6) | 215 (70.9) | 196 (69.0) | 173 (70.9) | 144 (72.0) |
| Methotrexate |  |  |  |  |  |  |  |
| Cohort 1, N (%) | 9 (1.5) | 38 (6.5) | 55 (10.2) | 70 (13.6) | 89 (18.6) | 92 (23.2) | 99 (26.0) |
| Cohort 2, N (%) | 128 (36.9) | 174 (52.7) | 167 (54.4) | 163 (53.8) | 151 (53.2) | 140 (57.4) | 111 (55.5) |
| bDMARD* |  |  |  |  |  |  |  |
| Cohort 1, N (%) | 0 (0) | 0 (0) | 0 (0) | 0 (0) | 0 (0) | 1 (0.3) | 10 (2.6) |
| Cohort 2, N (%) | 2 (0.6) | 3 (0.9) | 14 (4.6) | 16 (5.3) | 20 (7.0) | 22 (9.0) | 29 (14.5) |
| Oral Steroids |  |  |  |  |  |  |  |
| Cohort 1, N (%) | 50 (8.1) | 76 (13.0) | 82 (15.2) | 84 (16.3) | 74 (15.5) | 57 (14.4) | 56 (14.7) |
| Cohort 2, N (%) | 80 (23.1) | 75 (22.7) | 65 (21.2) | 60 (19.8) | 58 (20.4) | 44 (18.0) | 29 (14.5) |

*Percentages are given as a percentage of the number of patients in the corresponding cohort at the corresponding follow-up*

*bDMARD = biologic disease modifying antirheumatic drug, N = number, sDMARD = synthetic disease modifying antirheumatic drug*

** sDMARDs included: gold, penacillamine, sulphasalazine, (hydroxy)chloroquine, methotrexate, azathioprine, cyclophosphamide, leflunominde. bDMARDs included: etanercept, infliximab, adalimumab, rituximab*
